# A pediatric patient with Warsaw breakage syndrome presenting with epilepsy: a case report and literature review

**DOI:** 10.3389/fnins.2026.1751535

**Published:** 2026-03-03

**Authors:** Yixuan Zhang, Zhi Yi, Ying Zhang, Zhenfeng Song, Chengqing Yang, Fei Li, Kaixuan Liu, Jiashuo Li, Jiao Xue

**Affiliations:** 1 School of Medicine, Qingdao University, Qingdao, China; 2 Department of Pediatric Neurology, The Affiliated Hospital of Qingdao University, Qingdao, Shandong, China

**Keywords:** cohesinopathy, cranial magnetic resonance imaging, DDX11, epilepsy, Warsaw breakage syndrome

## Abstract

**Background:**

Warsaw breakage syndrome (WABS) is a rare disease caused by mutations in the DDX11 gene. It is characterized by severe growth restriction, microcephaly, and sensorineural hearing loss, and reports of coexisting epilepsy are even rarer. There are no studies on the focused synthesis of epilepsy phenotypes in WABS.

**Methods:**

A clinical review is conducted for a patient diagnosed with WABS. And a comprehensive search is performed using PubMed, Web of Science, and Scopus. We select only papers that report patients with WABS and epilepsy.

**Results:**

We present a boy exhibiting the core manifestations of this syndrome. In addition to growth restriction, microcephaly, and sensorineural hearing loss, he has experienced recurrent epileptic seizures since 7 months of age. The child showed resistance to multiple antiepileptic drugs, with seizure types progressing from focal to epileptic spasms. Whole-exome sequencing identified two variants in the patient’s DDX11 gene: c.2120delT (p.F707Sfs*60) and c.1949-3C>T (splicing). A literature review identified a total of 7 previously reported children with WABS complicated by epilepsy, and we collected and summarized their clinical and genetic information.

**Conclusion:**

We report a child with WABS whose main symptom was epilepsy. This case expands the known mutation spectrum of WABS and provides a comprehensive summary of clinical and genetic data for WABS patients presenting with epilepsy.

## Introduction

First described by van der Lelij et al., WABS is a rare autosomal recessive disorder caused by mutations in the DDX11 gene, and has since been recognized as a distinct genomic instability syndrome (Bharti et al., 2014; van der Lelij et al., 2010). DDX11 encodes a DNA helicase essential for sister chromatid cohesion and chromosome segregation during cell division (Shah et al., 2013). Loss of functional DDX11 helicase disrupts sister chromatid cohesion and DNA repair. This disruption causes chromosomal instability, which will be clinically manifested as growth and developmental abnormalities and other characteristics (van Schie et al., 2020). Despite being identified over a decade ago, its full clinical and molecular spectrum remains poorly characterized due to its rarity and phenotypic variability, with limited reported mutations and insufficient clinical data precluding a clear genotype–phenotype correlation for DDX11 mutations.

Growth retardation, microcephaly, and sensorineural hearing loss constitute the classic WABS triad. Beyond these core features, WABS is associated with a range of additional phenotypes, including genitourinary system abnormalities, cardiac defects, structural brain abnormalities, recurrent infections, abnormal skin lesions (van der Lelij et al., 2010; Alkhunaizi et al., 2018; Bailey et al., 2015; Bottega et al., 2019; Capo-Chichi et al., 2013). In recent years, an increasing number of clinical reports have identified seven children with WABS who also experience seizures.

## Methods

The patient was admitted to our department in October 2020. Clinical data were reviewed to obtain information. The patient underwent blood cell count, blood biochemistry, electrolyte analysis, cranial magnetic resonance imaging (MRI), video electroencephalography (EEG), and positron emission tomography-computed tomography (PET-CT) were performed. Whole-exome sequencing of peripheral blood samples from the patient and both parents was conducted using high-throughput sequencing technology. Written consent was obtained from patient’s parents for publication of his clinical phenotype and genotype.

We conducted a comprehensive literature search in PubMed, Web of Science, and Scopus databases using the key terms “Warsaw Breakage Syndrome” OR “WABS” AND “epilepsy” OR “seizures.” Only studies reporting complete clinical data of WABS patients with concomitant epileptic seizures were included.

## Results

A male child with developmental delay was admitted to our hospital for intractable epilepsy. Since 7 months of age, he has experienced episodes of excessive salivation and involuntary twitching of the right eyelid without obvious triggers. These episodes occurred dozens of times per day, were unremitting, and were not associated with loss of consciousness. Cranial MRI revealed the local gyrus of the right temporal lobe was widened with abnormal signals, and multiple small nodules were found under the ependyma of the bilateral lateral ventricles. These findings are consistent with gray matter heterotopia. Video EEG result indicated an abnormal infant EEG. During video EEG monitoring, the child experienced one seizure while awake, and the EEG indicated a focal onset arising from the right cerebral region. In addition, during the interictal period, spikes, spike-and-waves, and sharp-and-waves were observed in the right frontal lobes, and anterior, middle, and inferior temporal lobes. Similar discharges, including poly-spikes, and spike-and-waves and sharp-and-waves, were detected in the left middle and posterior temporal lobes. The child was therefore diagnosed with focal epilepsy. Treatment with oxcarbazepine was initiated, but seizures persisted. And the response remained poor even after adding sodium valproate and levetiracetam. At 11 months of age, the patient’s seizures progressed to epileptic spasms. During each episode, the limbs jerked, and the seizures occurred in clusters, with 4–5 events per cluster and approximately 5–6 clusters per day. The EEG showed multifocal spikes, poly-spikes and spike waves discharges during the interictal period. A cluster of spasms were detected, and the ictal EEG showed clustered generalized slow waves with low-amplitude fast activities discharging for about 1 s (Supplementary Figure S1). According to the guidelines released in 2022, the child meets the diagnosis of infantile epileptic spasms syndrome (Zuberi et al., 2022). PET-CT showed reduced cortical glucose metabolism in multiple areas of the right frontal lobe. Levetiracetam was discontinued, and topiramate and high-dose methylprednisolone were initiated. After discontinuation of methylprednisolone, the child continued to use sodium valproate, oxcarbazepine, and topiramate. And the child’s seizures ceased.

**Figure 1:**
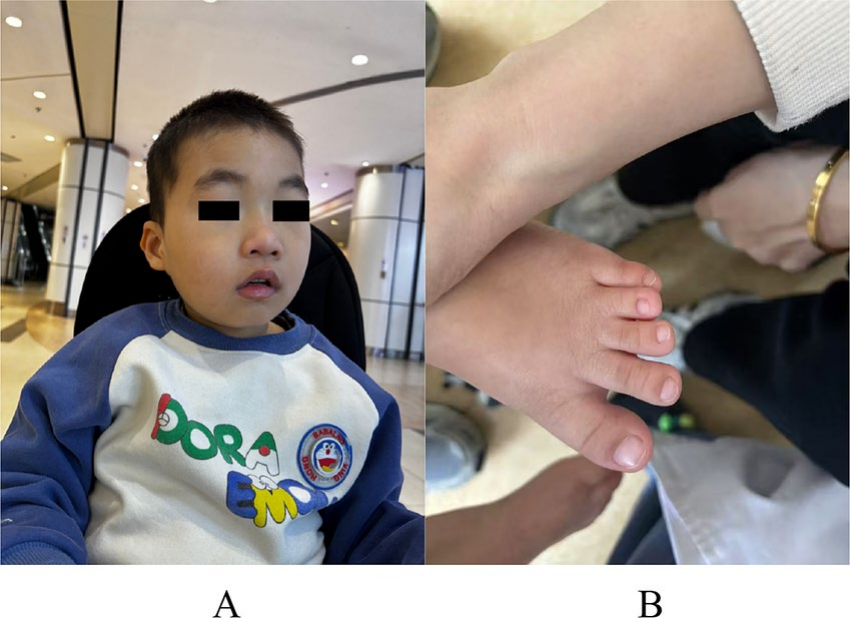
Distinctive facial and skeletal features of the patient. **(A)** Microcephaly, wide eye distance and external auditory canal malformation; **(B)** Syndactyly of the third and fourth toes.

The patient was followed for 4 years and experienced no further seizures. During this period, a brainstem auditory evoked potential examination showed no identifiable waveforms in the right ear. And the patient was diagnosed with right-sided hearing loss. The patient is now 5 years old, but his growth and developmental level remain markedly delayed compared with peers. The main manifestations are unstable walking, inability to stand on one foot, only a small amount of reduplicated words, and little active communication. In addition, we observed that the child’s face had special features, including microcephaly, wide eye distance, epicanthus, and external auditory canal malformation (Figure 1A). The child also had syndactyly of the third and fourth toes (Figure 1B).

Because the child had distinct facial features and refractory epileptic seizures, we performed whole-exome sequencing on peripheral blood samples from the child and his parents. Two variants are identified in the DDX11 gene of the patient: c.2120delT (p.F707Sfs*60) and c.1949-3C>T (splicing). The former, inherited from the father, is a single-nucleotide deletion that shifts the reading frame and introduces a premature stop codon after 59 amino acids. The latter, inherited from the mother, is a splicing mutation, which may disrupt normal mRNA processing and lead to abnormal protein products. According to the American College of Medical Genetics and Genomics (ACMG) guidelines, the first variant is classified as likely pathogenic (PVS1 + PM2), while the second is classified as a variant of uncertain significance (PM3). Although this variant has not been previously reported in the literature and lacks pathogenic annotation in Clinvar database, the DDX11 mutation together with the child’s clinical features support a diagnosis of WABS. In addition, whole-exome sequencing identified two additional variants that were classified as variants of uncertain significance according to the ACMG guidelines. One variant was a heterozygous KCNQ2 variant inherited from the father; although KCNQ2 is associated with benign familial neonatal seizures, the patient’s phenotype was not consistent with this disorder. The second variant was a heterozygous SETBP1 variant inherited from the mother; pathogenic variants in SETBP1 are known to cause autosomal dominant intellectual disability type 29 and Schinzel-Giedion syndrome, neither of which matched the patient’s clinical presentation. Therefore, these two variants were considered unlikely to be causative of the patient’s phenotype.

## Discussion

WABS is caused by biallelic pathogenic variants in the DDX11 gene, which encodes a DNA helicase essential for maintaining genome stability. Loss or severe reduction of DDX11 function impairs sister chromatid cohesion, particularly at the centromeric region. Under replication stress, this defect increases chromosome breakage and genomic instability (van Schie et al., 2020; Arroyo-Carrera et al., 2023). Such instability forms the biological basis of the clinical manifestations of WABS.

WABS is a rare genetic disorder, and cases in which epilepsy predominates are even rare. To date, there are no studies on the focused synthesis of epilepsy phenotypes in WABS. By reviewing previously published case reports, we identified seven patients diagnosed with WABS who exhibited epileptic manifestations, and their clinical features were summarized in Table 1. We find that, in addition to the classic triad of WABS, all seven patients exhibit facial dysmorphism and intrauterine growth restriction. This observation indicates that, in addition to assessing postnatal clinical features, careful evaluation of prenatal examination findings is equally important. Currently, chromosomal abnormalities, imbalance in umbilical artery-to-vein blood flow ratios, and maternal chronic diseases are recognized as major risk factors for intrauterine growth restriction (Frusca et al., 2018; Ohuma et al., 2023; Tsikouras et al., 2024). When fetal facial malformations and intrauterine growth restriction are detected, genetic testing should be considered. In addition to these clinical features, our patient also exhibited skeletal abnormalities as well as hypotonia. Moreover, among the seven reported WABS cases with epilepsy, two patients presented with structural brain abnormalities.

**Table 1:** Clinical characteristics of patients with WABS presenting with epilepsy.

Clinical phenotype	Patient 1	Patient 2	Patient 3	Patient 4	Patient 5	Patient 6	Our patient
Sex	F	M	M	M	F	M	M
Microcephaly	+	+	+	+	+	+	+
Sensorineural hearing loss	+	+	+	+	+	+	+
Developmental delay	+	+	+	+	+	+	+
Intrauterine growth restriction	+	+	+	+	+	+	+
Facial dysmorphia	+	+	+	+	+	+	+
Skin abnormalities	+	+	−	−	+	+	−
Skeletal abnormalities of fingers/toes	−	−	+	+	+	−	+
Cochlear hypoplasia	+	−	+	−	+	−	+
Brain abnormalities	−	−	+	−	+	−	+
Congenital hypothyroidism	−	−	−	+	+	−	−
Seizures (epilepsy)	+	+	+	+	+	+	+
Single palmar crease	−	−	−	−	−	−	−
Heart abnormalities	−	−	−	−	+	+	−
Genitourinary system abnormalities	−	−	−	−	−	+	−
Hypotonia	−	+	−	+	−	−	+

**Figure 2:**
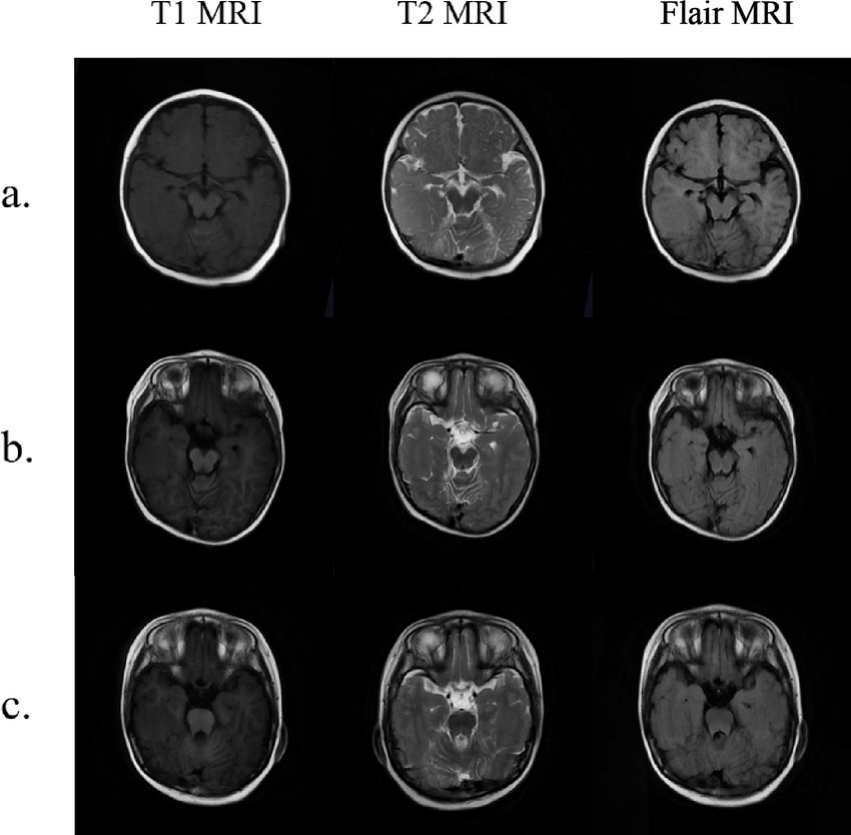
Axial MRI demonstrates widening of the right temporal lobe gyrus. **(a)** 7 months of age; **(b)** 2 years and 9 months of age; **(c)** 4 years and 8 months of age.

**Figure 3:**
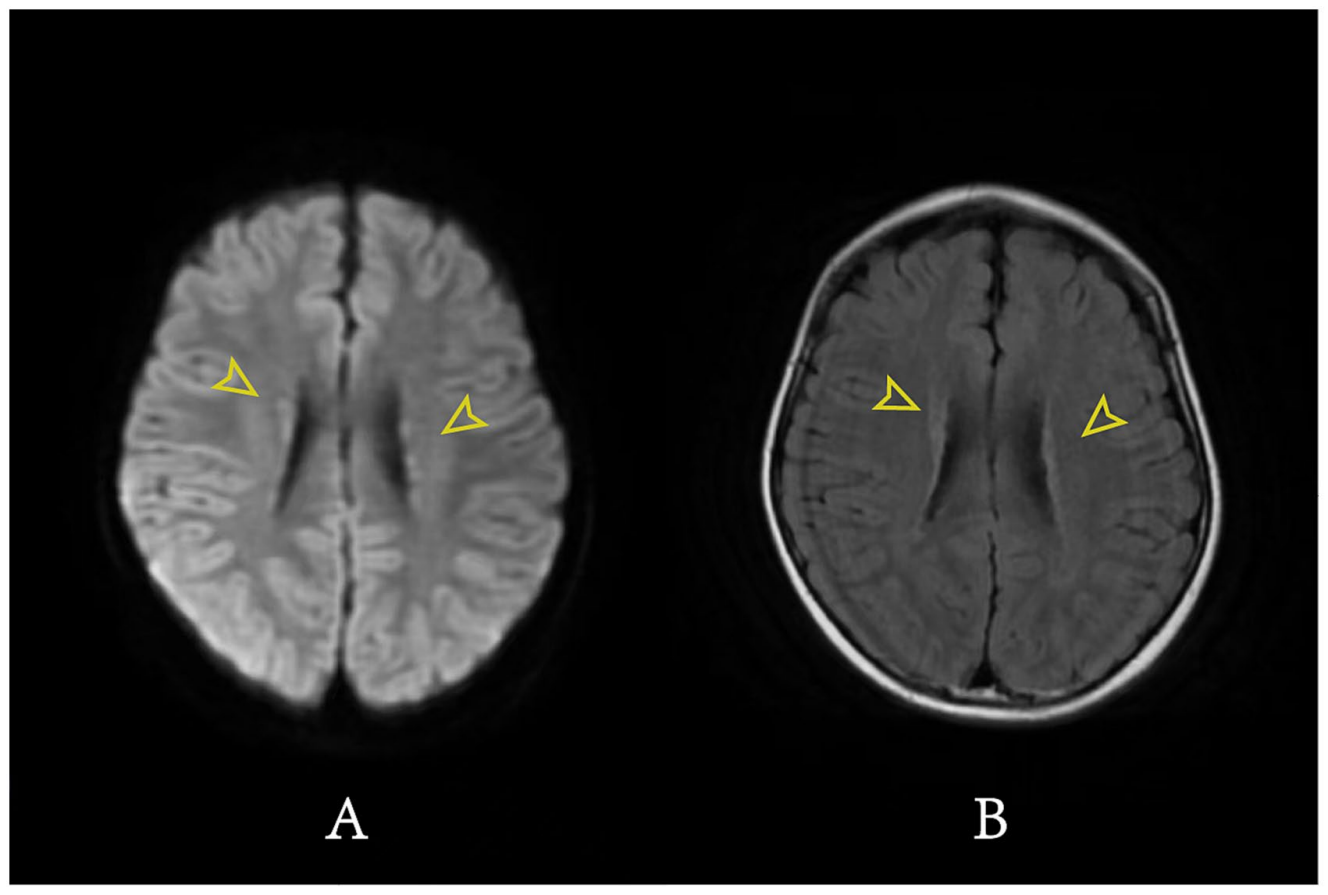
MRI demonstrates bilateral periventricular gray matter heterotopia. **(A)** DWI MRI; **(B)** T2 Flair MRI; Yellow arrows: bilateral periventricular gray matter heterotopia.

In this study, we summarize the seizure types, responses to antiepileptic treatment, and cranial MRI findings of WABS patients with epilepsy (Table 2). In WABS patients, the first seizure typically occurs during the preschool or early school-age period, with a median onset age of 70 months. Besides, the seizure types in these patients are diverse, including focal seizures and generalized seizures, and all reported patients experience some degree of impaired consciousness. Our case is the first to describe spasm seizures in a child with WABS. It adds a new seizure type to the known spectrum of this disorder. Reviewing previous studies, we find that not all epileptic seizures in WABS can be effectively controlled with medication. Alkhunaizi et al., (2018) reported a child with tonic-clonic seizures. The seizures were controlled with clonazepam alone. However, our patient required multiple antiepileptic drugs to control seizures. We reviewed the cranial MRI results of the two patients. The first patient had true microcephaly and simplified cerebral gyri. The second patient had these features and also gray matter heterotopia (Figures 2, 3). This additional structural abnormality may explain the failure of monotherapy with a single antiepileptic drug to control the seizures. Besides, the children reported by Arroyo-Carrera et al. (2023) showed only partial improvement after levetiracetam treatment, and still had frequent seizures. However, the small number of cases and limited clinical data prevent us from identifying the most effective antiepileptic drugs for children with different seizure types. Future studies should accumulate more clinical data to establish optimal antiepileptic treatment strategies for this subset of patients.

**Table 2:** Clinical information of WABS patients with epilepsy.

Case		Age of onset (months)	Form of onset	Imaging examination	Therapeutic drugs	Therapeutic effect
Janne et al.	WAB04	36	NA	NA	NA	NA
	WAB05	84	NA	NA	NA	NA
Alkhunaizi et al.		84	Tonic-clonic Seizures	Microcephaly vera with simplification of the gyri	Clonazepam	Effective
Rabin et al.	1	70	NA	NA	NA	NA
	2	108	Complex Partial Seizure	NA	NA	NA
Ignacio et al.		42	NA	NA	Levetiracetam	Moderate
Present study		7	Tonic-clonic Seizures	Widening of cerebral gyrus with gray matter ectopia	Topiramate	Moderate
					Oxcarbazepine	
					Depakine	

NA, Not available.

Our patient showed periventricular gray matter heterotopia, which may have contributed to the refractory epilepsy. Periventricular gray matter heterotopia is a neuronal migration disorder in which neurons fail to reach the cerebral cortex and instead form nodules along the lateral ventricular walls (Barkovich et al., 2012). These nodules contain abnormal pyramidal neurons and reactive astrocytes. Previous studies have shown that ectopic neurons within heterotopic nodules exhibit increased expression of the voltage-gated sodium channel, which lowers the action potential threshold and increases neuronal excitability (Di Matteo et al., 2025). Importantly, epilepsy associated with periventricular gray matter heterotopia is not thought to arise from a single heterotopic nodule. Instead, seizures appear to result from the combined involvement of multiple heterotopic nodules and the surrounding cerebral cortex. Adjacent cortical regions therefore also play a role in seizure generation and propagation (Watrin et al., 2015).

DDX11 encodes a DNA helicase required for genome stability during cell division, a process that is critical for normal neurodevelopment. Disruption of genome integrity during early brain development may affect neuronal proliferation and migration. However, no studies to date have demonstrated a direct mechanistic role of DDX11 in neuronal migration. Although our patient carried biallelic variants in DDX11 and presented with periventricular gray matter heterotopia, a direct causal relationship cannot be established based on a single case. At present, there is insufficient evidence to confirm an association between DDX11 mutations and gray matter heterotopia. Further well-characterized cases from different populations will be necessary to clarify this potential genotype-phenotype relationship.

We summarize the genetic information of the seven children (Table 3). Most children have compound heterozygous mutations, while two have homozygous mutations. In addition, both missense and splicing site mutations can make the clinical manifestations and ACMG typing classified as pathogenic. Our patient has developed a new variant of uncertain significance. We used RNA Splicer software to assess the potential splicing impact of the c.1949-3C>T variant. This variant is located in the intronic region downstream of exon 19. In silico prediction suggested that the variant may generate an abnormal splice acceptor site. This could lead to aberrant splicing, in which the 18 base pairs upstream of exon 20 are misidentified as intronic and removed. After introduction of the variant, the DanQ score increased from 0.8019 to 0.9369, indicating an increased likelihood that this position functions as a splice acceptor site. However, as this evidence is based solely on computational prediction, the functional consequences of this variant remain to be experimentally confirmed. At present, the c.1949-3C>T variant is predicted to potentially impair DDX11 gene function. However, its pathogenic significance requires further functional validation.

**Table 3:** Genetic information of children with WABS presenting with epilepsy.

Case		Gene	Variant 1		Variant 2		Inheritance and zygosity	ACMG classification	Criteria applied
			DNA	Protein	Nucleotide	Amino acid			
Janne et al.	WAB04	DDX11	c.1930G>A	p.V644M	c.2114G>A	p.C705Y	Compound heterozygous variation	Pathogenic	NA
	WAB05	DDX11	c.2571C>A	p.S857R	c.1672C>T	p.R558*	Compound heterozygous variation	Pathogenic	PVS1
									PM2
									PM3
Alkhunaizi et al.		DDX11	c.606delC	p.Y202*	c.2372G>A	p.R791Q	Compound heterozygous variation	Pathogenic	NA
Rabin et al.	1	DDX11	c.1763-1G>C	Splice site	c.1763-1G>C	Splice site	Homozygous variation	Pathogenic	NA
	2	DDX11	c.1763-1G>C	Splice site	c.1763-1G>C	Splice site	Homozygous variation	Pathogenic	NA
Arroyo-Carrera et al.		DDX11	c. 1403dup	p.S469Vfs*32	c.2371C>T	p.R791W	Compound heterozygous variation	Pathogenic	PVS1
									PM2
									PM3
Present study		DDX11	c.2120delT	p.F707Sfs*60	c.1949-3C>T	Splice site	Compound heterozygous variation	Likely pathogenic/uncertain	PVS1
									PM2
									PM3

NA, Not available.

Notably, the child’s mother became pregnant again, and we performed whole-exome sequencing on DNA extracted from amniotic fluid. The result indicate that the fetus carries the same DDX11 mutation as the affected child, which is likely pathogenic. Ultimately, the mother opted for termination of the pregnancy.

Although epilepsy is not a common manifestation of WABS, the syndrome should be strongly suspected in children with epilepsy who also present with microcephaly, growth retardation, and sensorineural hearing loss. And the timely genetic testing is recommended to confirm the diagnosis. However, previous studies have indicated that antiepileptic drug treatment is not very effective in children with WABS. Identifying the optimal treatment strategy to control seizures is important for improving their quality of life. However, not all patients with WABS present with epilepsy and clinical data are limited with no clear and effective antiepileptic treatment strategy to date. Further in-depth research is needed to address this gap.

## Conclusion

We report a child with WABS whose presenting symptom was epilepsy. This case expands the known genetic mutation spectrum of WABS and provides the comprehensive summary of the clinical and genetic features of children with WABS presenting with epilepsy. In children with epilepsy who present with the classic WABS triad (severe growth restriction, microcephaly, and sensorineural hearing loss), timely genetic testing is recommended to confirm the diagnosis.

## Data Availability

The data presented in this study are available through Clinvar (https://www.ncbi.nlm.nih.gov/clinvar/), with the following accession number SCV006311630.1. Further inquiries can be directed to the corresponding author.
