# Supplementary material for: A pediatric patient with Warsaw breakage syndrome presenting with epilepsy: a case report and literature review
Source: Front Neurosci. 2026 Mar 3;20:1751535. doi: 10.3389/fnins.2026.1751535 (PMC12992309; doi:10.3389/fnins.2026.1751535)
Supplement: Supplementary file 1 [file Data_Sheet_1.docx]

Supplementary Material

## Supplementary Figure


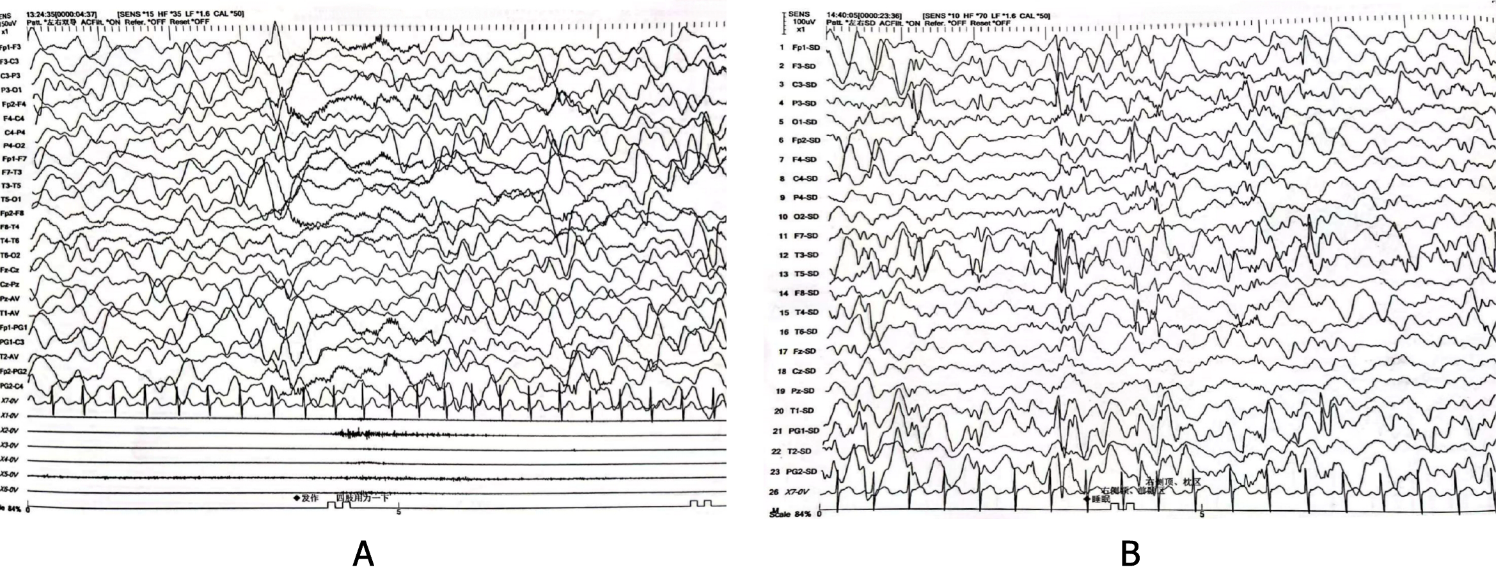


**Supplementary Figure.** **Electroencephalograms (EEG) supporting the diagnosis of i****nfantile epileptic spasms syndrome.** A: Clustered generalised slow waves with low-amplitude fast activities in the ictal period; B: Multifocal spikes, polyspikes and spike waves discharges in the interictal period.
